# Survival Trends in Patients Under Age 65 Years With Mantle Cell Lymphoma, 1995–2016: A SEER-Based Analysis

**DOI:** 10.3389/fonc.2020.588314

**Published:** 2020-10-20

**Authors:** Hongyu Wu, Jianwei Wang, Xuanye Zhang, Hang Yang, Yu Wang, Peng Sun, Qingqing Cai, Yi Xia, Panpan Liu

**Affiliations:** ^1^Department of Medical Oncology, Sun Yat-sen University Cancer Center, Guangzhou, China; ^2^State Key Laboratory of Oncology in South China, Guangzhou, China; ^3^Collaborative Innovation Center for Cancer Medicine, Guangzhou, China

**Keywords:** mantle cell lymphoma, non-Hodgkin lymphoma, survival, SEER program, immunotherapy

## Abstract

**Purpose:** The treatment paradigm for mantle cell lymphoma (MCL), a B-cell malignancy, has shifted considerably during the past decades. This study aimed to evaluate time trends in overall survival (OS) and disease-specific mortality (DSM) of younger (age ≤ 65 years) patients with MCL from 1995 to 2016.

**Methods:** We used the Surveillance, Epidemiology, and End Results database. Year of diagnosis was divided into three eras: the chemotherapy-alone era (1995–2000), intensified-immunochemotherapy era (2001–2012), and targeted-therapy era (2013–2016). We used the Kaplan–Meier method, log-rank test, and subdistribution proportional hazard regression in the analysis.

**Results:** A total 4,892 patients were identified. Median OS increased from 67 months in the chemotherapy-alone era to 107 months in the intensified-immunochemotherapy era (*P* < 0.001). The DSM rate decreased significantly from 1995 to 2016 (*P* < 0.001); the adjusted hazard ratios of MCL-specific death were 0.589 (*P* < 0.001) for the intensified-immunochemotherapy era and 0.459 (*P* < 0.001) for targeted-therapy era, as compared with the chemotherapy-alone era. Patients with advanced-stage MCL exhibited lowering risk of death across the three eras (*P* < 0.001).

**Conclusions:** During 1995–2016, survival in younger patients with MCL increased significantly, especially those with advanced-stage disease, potentially reflecting the impact of advancement in treatment modalities on MCL outcome.

## Introduction

First adopted as an official entity in 1994 (1), mantle cell lymphoma (MCL) is a relatively rare B-cell non-Hodgkin lymphoma (NHL) accounting for about 5–7% of NHL (2). MCL has an increasing incidence overall and has a morbidity of 1–2/100,000 in recent decades. Approximately three-quarters of patients are male. Most patients present with advanced-stage disease at diagnosis (3, 4). Primary presentation of extranodal disease is found in about 25% of patients. Common extranodal sites of involvement include the gastrointestinal tract, breast, pleura, and orbit. MCL is genetically characterized by the translocation t(11;14)(q13;q32) and leads to overexpression of cyclin D1 (5, 6). Most patients with this disease present with an aggressive clinical course and require treatment.

Treatment options are mainly based on age (under or over age 65 years) and comorbidities (4). Our study focused on survival trends among younger patients with MCL (age ≤ 65 years) as they are healthier than their older counterparts and have less treatment-related complications, and can therefore better reflect the shift in treatment strategies. Before 2000, CHOP-based induction chemotherapy (cyclophosphamide, doxorubicin, vincristine, prednisone) was the standard treatment for younger patients with MCL (7). The rate of complete response (CR) with the standard CHOP regimen was low, and median survival was in the range of 2–5 years. During 2001–2012, intensified immunochemotherapy regimens containing rituximab and high-dose cytarabine (HDAC) followed by consolidative autologous stem cell transplantation (ASCT) provided the first breakthrough in clinical management of aggressive MCL by improving the response quality and duration in younger patients. The regimens include alternating R-CHOP/R-DHAP (rituximab, dexamethasone, high-dose cytarabine, and cisplatin), the Nordic MCL2 protocol (rituximab with dose-escalated cyclophosphamide and doxorubicin, vincristine, prednisone [R-maxi-CHOP] alternated with HDAC), the MD Anderson protocol (hyperfractionated cyclophosphamide, vincristine, doxorubicin, dexamethasone alternating with high-dose methotrexate and cytarabine with rituximab [R-hyper-CVAD/MA]), and deliver median overall survival (OS) over 10 years (8–13). However, such therapies did not represent a curative approach and were associated with acute and long-term toxicity. During 2013–2016, novel agents, led by Bruton's tyrosine kinase (BTK) inhibitors along with other oral agents such as lenalidomide, bortezomib, temsirolimus, and venetoclax, which are generally well-tolerated and effective, represented the second wave of a clinical revolution that has significantly improved treatment options and outcome among patients with MCL (14, 15). Using chemo-free induction will mitigate toxicities and the risk of second cancers, which are associated with the use of intensive chemoimmunotherapy regimens in these patients.

The effect of these protocols and agents was confirmed in a series of clinical trials. However, because of the stringent eligibility criteria for clinical trials, patients with less severe disease and no complications were more likely to be selected for inclusion in these trials, limiting the generalizability of the conclusions. What's more, previous clinical trials have focused on a specific treatment regimen and thus could not predict the overall survival trends in the whole population. Owing to these limitations, a study based on the general patient population can more practically estimate the effect of new agents and regimens. Prior studies using Surveillance, Epidemiology, and End Results (SEER) data have analyzed the impact of changes in the treatment paradigm on survival trends in MCL; however, these studies did not properly consider the era of targeted therapies (16–19). A study based on the general patient population can help to identify how the new agents and regimens affect survival in the real world. Under these conditions, we sought to prove our hypothesis that survival in younger patients has increased over successive periods (or eras) representing the respective primary clinical management of MCL.

## Materials and Methods

### Data Source

The included patient data were derived from the SEER program, an ongoing project of the National Cancer Institute of the National Institutes of Health. The SEER registry contains ~30% of the population of the United States (US) (20) and includes data that can be traced back to 1973. Various crucial information can be found in the SEER database, such as cancer diagnosis, patient demographics (age, ethnicity), survival time, and cause of death (21).

### Study Population

Patients recorded in SEER were eligible for this study if they were diagnosed with MCL between January 1, 1995 and December 31, 2016 and were age 65 years or younger. The diagnosis for MCL was in line with the International Classification of Disease for Oncology, 3rd Edition (ICD-O-3) code in SEER. Cases were excluded if survival time was unknown (*n* = 4). A total 4,892 patients were finally included in the analysis.

### Study Variables

The era of diagnosis was the main variable, with three categories distinguished according to the representative drugs during each era. Cases from 1995 to 2000 represented treatment with chemotherapy alone; those from 2001 to 2012 were representative of intensive immunochemotherapy; and cases from 2013 to 2016 were treated as a proxy for targeted therapy. Covariates including age at diagnosis, sex, tumor stage, and ethnicity were introduced, to adjust the hazard ratio (HR).

Survival outcome variables taken into consideration were survival time and status. In SEER, survival time is counted from the date of diagnosis to the date of last contact for patients not known to have died (20). Status was deduced from consideration of the SEER cause-specific death classification, and classified as MCL-specific death, non-MCL death, and alive. In that case, competing risks identified as non-MCL cause of death were adjusted in the analysis.

### Statistical Methods

Clinical characteristics were compared using the Pearson χ^2^ test in that the independent variables considered were all unordered categorical variables. The overall survival for younger patients with MCL in each era was estimated using the Kaplan–Meier method and compared with the log rank test. The cumulative incidence function allowed for the estimation of MCL-specific mortality (22). Gray's test was applied to compare MCL-specific mortality. HRs and 95% confidence intervals (CIs) for the collected variables were computed at both univariate and multivariate levels using the subdistribution hazard function (22–24). To verify stability of the results, we also carried out a subgroup analysis based on tumor stage using a cause-specific hazard model (25). The precondition of satisfying the proportional hazard assumption for both hazard models was confirmed. To account for the uneven distribution of patients in term of age intervals among the three treatment groups, multivariate proportional hazards regression was used to reduce potential confounding bias. Values were regarded as statistically significant with *P* < 0.05. All statistical analysis was conducted using *R* x 64 3.6.1 (The *R* Project for Statistical Computing, Vienna, Austria) and Stata/SE 12.0 (StataCorp LLC, College Station, TX, USA).

## Results

### Clinical Features

Clinical features in the indicated eras are summarized in Table 1. Of 4,892 patients who were eligible for analysis, 571 were diagnosed between 1995 and 2000, 3,073 were diagnosed between 2001 and 2012, and 1,248 were diagnosed between 2013 and 2016. In this study, 2,125 (43.4%) patients were between 50 and 59 years of age and 1,962 (40.1%) were between 60 and 65 years of age; 3,617 (73.9%) patients were men, 3,677 (75.2%) were in advanced stages, and 3,841 (78.5%) were non-Hispanic white. A chi-square test was performed to compare the baseline characteristics over time. Clinical features including age (*P* < 0.001), stage (*P* < 0.001), and ethnicity (*P* = 0.008) showed significant variations over the different periods; there was little variation according to sex (*P* = 0.526).

**Table 1:** Clinical features of younger patients with mantle cell lymphoma in the indicated eras.

	**Era**				
**Clinical features**	**1995–2000**	**2001–2012**	**2013–2016**	**Total**	P^*^
	**(*N* = 571)**	**(*n* = 3,073)**	**(*n* = 1,248)**	**(*n* = 4,892)**	
Age *N*(%)					<0.001
<50	146 (25.6%)	501 (16.3%)	158 (12.7%)	805 (16.5%)	
50–59	234 (41.0%)	1,361 (44.3%)	530 (42.5%)	2,125 (43.4%)	
60-65	191 (33.5%)	1,211 (39.4%)	560 (44.9%)	1,962 (40.1%)	
Sex *N*(%)					0.526
Female	160 (28.0%)	793 (25.8%)	322 (25.8%)	1,275 (26.1%)	
Male	411 (72.0%)	2,280 (74.2%)	926 (74.2%)	3,617 (73.9%)	
Stage *N*(%)					<0.001
Early	130 (22.8%)	433 (14.1%)	103 (8.3%)	666 (13.6%)	
Advanced	400 (70.1%)	2,459 (80.0%)	818 (65.5%)	3,677 (75.2%)	
Unknown	41 (7.2%)	181 (5.9%)	327 (26.2%)	549 (11.2%)	
Race *N*(%)					0.008
Non-hispanic white	462 (80.9%)	2426 (78.9%)	953 (76.4%)	3,841 (78.5%)	
Non-hispanic black	39 (6.8%)	174 (5.7%)	71 (5.7%)	284 (5.8%)	
Hispanic	37 (6.5%)	324 (10.5%)	158 (12.7%)	519 (10.6%)	
Other	33 (5.8)	149 (4.8%)	66 (5.3%)	248 (5.1%)	

* *Chi-square test was performed to compare the clinical features over time*.

### Median OS, 3-Year and 5-Year Mortality

**Figure 1:**
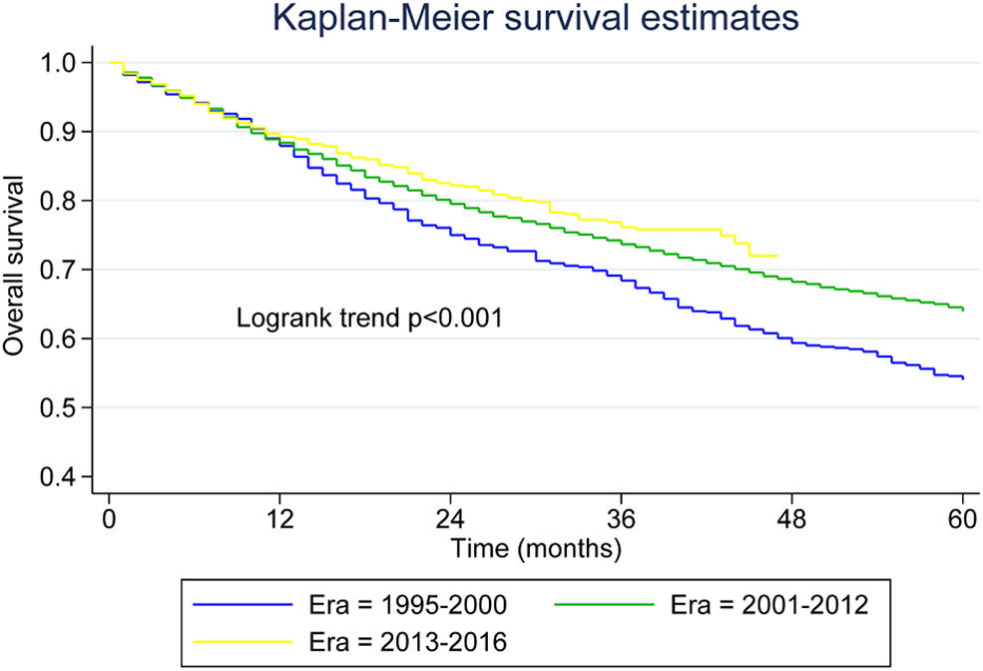
Kaplan–Meier survival curves for younger patients with mantle cell lymphoma across the three eras.

**Figure 2:**
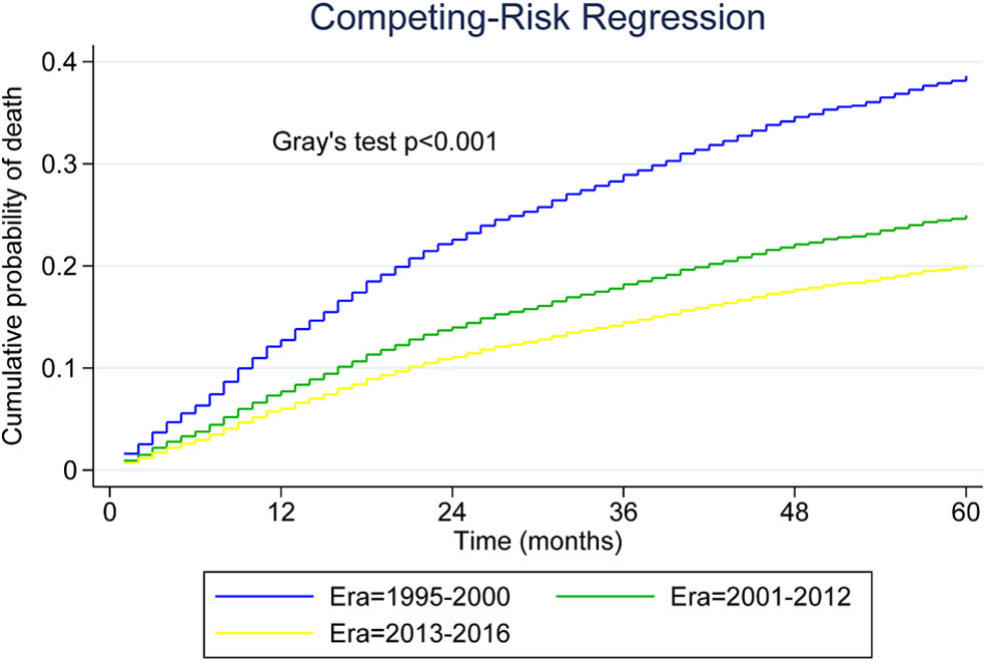
Five-year cumulative incidence of mantle cell lymphoma (MCL)-specific death for younger patients with MCL.

Table 2 presents median overall survival months, overall survival, and MCL-specific mortality. Figures 1, 2 show the survival curves and MCL-specific mortality of younger patients with MCL, respectively. The median overall survival for patients diagnosed from 1995 to 2000 was 67 months; this was 107 months for patients diagnosed between 2000 and 2012. Median survival time for patients diagnosed between 2013 and 2016 cannot yet be determined. It can be observed that the 3-year overall survival increased from 0.676 to 0.750 from the first to the third era (*P* = 0.012) and the 5-year overall survival increased from 0.534 to 0.631 from the first to the second era (*P* < 0.001). As for MCL-specific mortality, the 3-year mortality decreased from 0.261 to 0.180 from the first to the third era (*P* < 0.001) and the 5-year mortality decreased from 0.370 to 0.264 from the first to the second era (*P* < 0.001).

**Table 2:** Median overall survival (OS), 3-year and 5-year OS, 3-year and 5-year disease-specific mortality (DSM) in the indicated eras.

**Survival statistics**	**Era**				
	**1995–2000**	**2001–2012**	**2013–2016**	**Total**	***P***
	**(*N* = 571)**	**(*n* = 3,073)**	**(*n* = 1,248)**	**(*n* = 4,892)**	
Total cases, *N*	571	3,073	1,248	4,892	-
Death cases, *N* (%)	433 (75.8%)	1,506 (49.0%)	216 (17.3%)	2,155 (44.1%)	<0.001^a^
Median OS (months, 95% CI)	67 (58.833 ~ 75.167)	107 (98.767 ~ 115.233)	NA	99 (92.299 ~ 105.701)	-
3-year OS (95% CI)	0.676 (0.637–0.715)	0.727 (0.711–0.743)	0.750 (0.717–0.783)	0.724 (0.710–0.738)	0.012^b^
5-year OS (95% CI)	0.534 (0.500–0.568)	0.631 (0.613–0.649)	NA	0.621 (0.605–0.637)	<0.001^b^
3-year DSM (95% CI)	0.261 (0.225–0.297)	0.198 (0.184–0.212)	0.180 (0.151–0.209)	0.202 (0.190–0.214)	<0.001^c^
5-year DSM (95% CI)	0.370 (0.330–0.410)	0.264 (0.248–0.280)	NA	0.274 (0.260–0.288)	<0.001^c^

a *Overall survival was tested by log-rank test*.

b *Trend of 3-year and 5-year OS was tested using log-rank test*.

c *Trend of 3-year and 5-year MCL-specific cumulative incidence was tested using Gray's test*.

*NA means it hasn't been reached so far*.

### Univariate and Multivariate Analysis

The results of univariate and multivariate analysis are summarized in Table 3. Univariate analysis showed that the HR (95% CI) of MCL-specific mortality was 0.646 (0.570–0.731, *P* < 0.001) for the intensified-immunochemotherapy era and 0.513 (0.419–0.629, *P* < 0.001) for the targeted-therapy era, in comparison with the chemotherapy-alone era. In the multivariate analysis, the adjusted HRs (95% CI) were 0.589 (0.519–0.667, *P* < 0.001) for the intensified-immunochemotherapy era and 0.459 (0.374–0.564, *P* < 0.001) for the targeted-therapy era. The HRs were 1.240 (*P* = 0.005) for patients age 50 to 59 years and 1.332 (*P* < 0.001) for those age 60 to 65 years, as compared with patients age under 50 years. With non-Hispanic black patients set as the reference group, HRs were 1.173 (*P* = 0.235) for Hispanic patients, 0.924 (*P* = 0.479) for non-Hispanic white patients, and 0.865 (*P* = 0.381) for other ethnicities. In comparison with patients in early stages, the HRs for patients with advanced tumor stage were 1.739 (*P* < 0.001) and 1.157 (*P* = 0.282) for patients in other stages. Male patients had a 1.280-fold greater risk of dying from MCL than female patients.

**Table 3:** Univariate and multivariate analysis of clinical parameters associated with overall survival in younger patients with MCL.

**Variable**	**Univariate analysis**		**Multivariate analysis**	
	**Hazard ratio(95% CI)**	***P***	**Hazard ratio(95% CI)**	***P***
**Year of diagnosis**				
1995–2000	Reference	-	Reference	-
2001–2012	0.646 (0.570–0.731)	<0.001	0.589 (0.519–0.667)	<0.001
2013–2016	0.513 (0.419–0.629)	<0.001	0.459 (0.374–0.564)	<0.001
**Age, years**				
<50	Reference	-	Reference	-
50–59	1.171 (1.009–1.359)	0.038	1.240 (1.067–1.441)	0.005
60–65	1.240 (1.066–1.442)	0.005	1.332 (1.144–1.550)	<0.001
**Race**				
Non-hispanic black	Reference	-	Reference	-
Hispanic	1.166 (0.899–1.512)	0.248	1.173 (0.901–1.528)	0.235
Non-hispanic white	0.954 (0.767-1.185)	0.668	0.924 (0.741-1.151)	0.479
Other	0.871 (0.629-1.206)	0.405	0.865 (0.624-1.197)	0.381
**Stage**				
Early stage	Reference	-	Reference	-
Advanced stage	1.628 (1.384–1.915)	<0.001	1.739 (1.479–2.045)	<0.001
Other	1.100 (0.845–1.432)	0.479	1.157 (0.887–1.508)	0.282
**Sex**				
Female	Reference	-	Reference	-
Male	1.280 (1.130–1.449)	<0.001	1.279 (1.129–1.448)	<0.001

### Sensitivity Analysis

**Figure 3:**
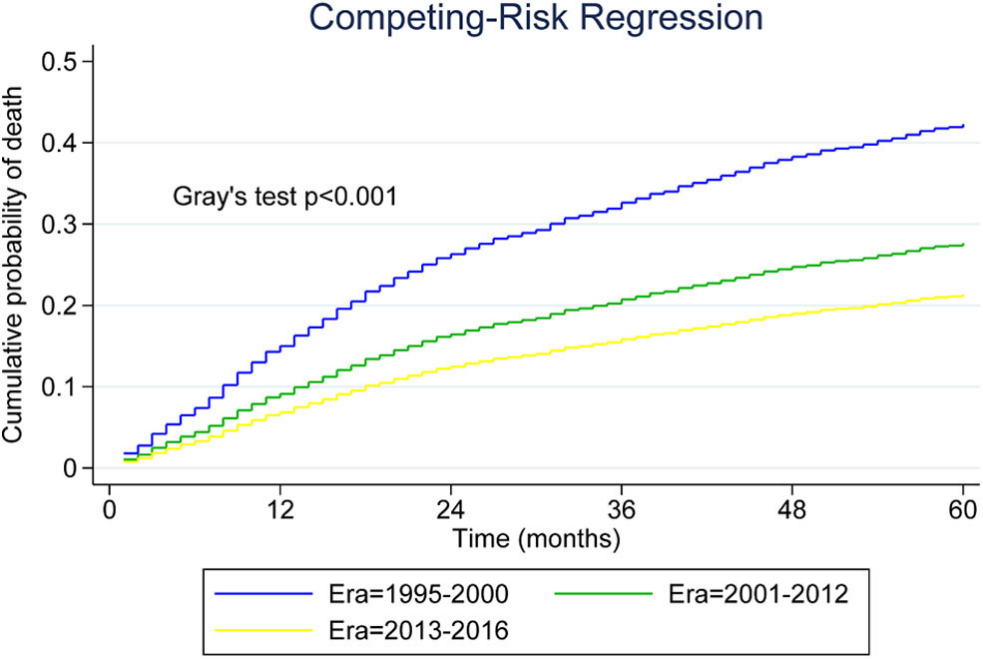
Five-year cumulative incidence of mantle cell lymphoma (MCL)-specific death for younger patients with advanced-stage MCL.

As shown in Figure 3, we plotted curves of MCL-specific mortality for patients with advanced-stage tumor. Using Gray's test, we observed a significant decrease across the three eras among patients with advanced-stage MCL (*P* < 0.001). Analysis was also conducted for patients with limited-stage MCL, and the results also indicated a decreasing trend (*P* < 0.001).

As presented in Table 4, we conducted multivariate analysis among patients in different stages. When regarding the chemotherapy-alone era as reference, the HRs were 0.588 (*P* < 0.001) for the intensified-immunochemotherapy era and 0.437 (*P* < 0.001) for the targeted-therapy era in patients with advanced-stage tumor. For patients with limited tumor stage, the HRs were 0.483 (*P* < 0.001) for the intensified-immunochemotherapy era and 0.733 (*P* = 0.348) for the targeted-therapy era; this indicated that only patients with advanced tumor stage had a lower risk of MCL-specific death after adjusting the covariates.

**Table 4:** Hazard ratio for year of diagnosis in different stage.

**Year of diagnosis**	**Hazard ratio (95% CI)**			
	**Advanced stage**	***P***	**Early stage**	***P***
1995–2000	Reference	-	Reference	-
2001–2012	0.588 (0.512–0.677)	<0.001	0.483 (0.347–0.673)	<0.001
2013–2016	0.437 (0.349–0.547)	<0.001	0.733 (0.382–1.403)	0.348

Two types of hazard functions are mainly used in the presence of competing risk. To verify stability of the results obtained using the subdistribution hazard function, we applied the cause-specific hazard function to build the model. Using the multivariate cause-specific hazard model, the adjusted HRs were 0.604 (*P* < 0.001) for the intensified-immunochemotherapy era and 0.519 (*P* < 0.001) for the targeted-therapy era, as compared with the chemotherapy-alone era. These results were similar to those obtained in the subdistribution hazard model.

## Discussion

We carried out the present population-based study to examine the survival trends in younger patients with MCL diagnosed during 1995–2016. To our knowledge, this is the first retrospective study covering the era of targeted therapy to analyze survival in younger patients with MCL. The results of our study indicate that survival in younger patients with MCL has improved significantly across the three periods investigated. The 5-year overall survival increased from 0.534 (in the first era) to 0.631 (second era) and the median overall survival increased from 67 months (first era) to 107 months (second era); overall survival in the third era cannot yet be determined. When we regress a multivariate subdistribution hazards function, the effect of distinct eras of diagnosis on the incidence of MCL-specific death was estimated as 0.589 (*P* < 0.001, second era) and 0.459 (*P* < 0.001, third era) compared with the first era, which showed a significant impact on mortality.

The significantly increased survival in the second era is likely attributable to the introduction of rituximab, high-dose cytarabine-containing regimens and ASCT consolidation. As our understanding of the molecular biology of MCL improves, the emergence of multiple targeted drugs, with BTK inhibitors such as ibrutinib leading the way, changes the modes of therapy in patients with MCL and presents as another phase of epocal progress. Ibrutinib was the first oral targeted agent for MCL approved by the US Food and Drug Administration (FDA), with single agent activity of 68% overall response rate (ORR), 21% CR rate, 13 months median progression-free survival (PFS) and uncommon grade 3 and 4 adverse events in relapsed or refractory MCL, thus providing patients the opportunity for treatment with less intensive and more effective regimens (14, 26). Later, the ibrutinib-combining chemo-free regimens also showed high activity, among which the combination of ibrutinib and rituximab has achieved 88% ORR, 44% CR, and 43 months median PFS (27). Furthermore, novel BTK inhibitors such as acarabrutinib and zanubrutinib show higher selective activity in relapsed MCL and have gradually been introduced into clinical practice (28, 29). Although not approved as single agent for relapsed and refractory MCL by the FDA, venetoclax, a BCL2 inhibitor, is considered a valuable agent. In a phase 2 study, the dual targeting of BTK and BCL2 with ibrutinib and venetoclax achieved a complete response rate of 44% at week 16, which was 9% higher than the ibrutinib monotherapy historical controls at the same time point (30). Recently, the ibrutinib-based combination has been evaluated in frontline settings in a number of studies and has produced a higher minimal residual disease (MRD)-negative remission rate, which is an important indication for long-term survival. In the results of the WINDOW 1 (NCT 02427620) chemo-free part 1 trial, the ORR was 100% (88% CR) in patients younger than age 65 years (31). In the phase 1/2 (OASIS, NCT 02558816) step C trial, a combination of ibrutinib, venetoclax, and obinutuzumab showed an ORR of 100% in 15 treatment-naïve patients when assessed at the end of cycle 2. In terms of MRD status, eight patients (others are ongoing) were assessed at the end of cycle 3 and all were MRD negative in BM (*n* = 6) and/or blood (*n* = 8) (32). In sum, MCL treatment is becoming focused on incorporating non-chemotherapeutic agents in the frontline setting, in the hopes of minimizing or even replacing chemotherapy.

The findings of our study were consistent with those of previous reports for the entire cohort of patients with MCL, based on population analysis. A cohort study conducted by Chandran et al. indicated that patients with MCL diagnosed between 2000 and 2007 had better predicted survival than those diagnosed before 2000, when adjusting potential confounders (17). Fu et al. confirmed the increasing survival in patients with MCL during 1995–2013, which reflected developments in treatment after 2000 (19). What's more, a study by Epperla et al. found continuous survival improvement in patients with MCL from 2000 to 2013, which also confirmed the effect of introduction of rituximab and novel agents; however, only a limited period was investigated (18). These studies included patients diagnosed before 2013 and therefore cannot properly reflect the effect of targeted drugs on clinical outcome.

Sensitivity analysis according to tumor stage indicated that the survival trend was improved only in advanced stages. Similar results were also obtained in studies conducted by Fu et al. (19) and Chandran et al. (17). According to the European Society for Medical Oncology (ESMO) Clinical Practice Guidelines (4), a shortened conventional chemotherapy induction followed by consolidation radiotherapy is suggested in patients with limited stages, such that they are less influenced by the new agents and protocols. Furthermore, these patients have always been considered potentially curable, with a relatively good prognosis; thus, they are less likely to be observed to gain a significant increase in survival. Sensitivity analysis of different types of hazard function showed that the effect of each era on mortality was also identified using the cause-specific hazard function. As a covariate, ethnicity was found to be statistically non-significant at univariate and multivariate level. However, differences in ethnicity have been reported to affect outcomes of patients with MCL (33), such that we retained this variable in the multivariate analysis.

There are several strengths in this study. To our knowledge, this is the first retrospective study covering the era of targeted therapy to analyze survival trends in patients with MCL. Furthermore, we used the cumulative incidence function to estimate mortality, rather than the complement of the Kaplan–Meier survival function, in that upward bias could be found if we naively used the latter function in the presence of non-MCL mortality. In addition, when we compared the two frequently applied hazard functions, we chose the subdistribution hazard function rather than the cause-specific hazard function to build a regression model; as a result; the former is better suited for estimating actual risk and prognosis whereas the latter is preferable when the focus is on investigating the etiology of disease. Lastly, data recorded in SEER cover ~34.6% of the US population and include detailed patient information and survival outcomes (34).

There are also some limitations in this study. First, we were only able to obtain data before 2016 through SEER, such that the third era was limited to 2013–2016, when novel targeted therapies were used mainly in a relapsed/refractory setting. Targeted therapies for first-line evaluation and longer observation are required. Second, information regarding specific treatment was not available; therefore, the proportion of each protocol in the different eras is unclear. For example, if some of the MCL patients in group 2 treated with intensified immunochemotherapy survived beyond 2013 and relapsed, they could have been treated with targeted agents such as Ibrutinib and Revlimid. In such cases, those patients could have gained benefit from targeted therapy. Third, several covariates related to prognosis were introduced into the hazard function regression to adjust the baseline of each era; nevertheless, some of these involving prognostic indicators, such as TP53 aberrations and Ki-67 proliferation of the included patients, could not be obtained. Additionally, since this study analyzed MCL patients in a long time span of over 20 years, it is possible that, in addition to the introduction of new drug treatment during this period, other factors such as improvements in diagnostics and patient support care, and changes in socio-economic status might also have contributed to the improved survival. As such, caution should be exercised in the interpretation of the observed results.

## Conclusions

During 1995–2016, survival in younger patients increased significantly over the three eras representing distinct clinical treatment for MCL. Subgroup analysis according to tumor stage indicated that the survival trend improved only in the advanced stage. The effect in each era on survival was confirmed using both the subdistribution hazard function and cause-specific hazard function.

## Data Availability Statement

Publicly available datasets were analyzed in this study. This data can be found here: Surveillance, Epidemiology, and End Results (SEER) database (https://seer.cancer.gov/seerstat/).

## Author Contributions

Conceptualization: HW, JW, YX, and PL. Methodology: HW, JW, XZ, and PL. Software: HY, YX, and PS. Formal analysis: HW, JW, HY, and YW. Resources: YW, YX, and PL. Data curation: YX, QC, and PS. Writing—original draft preparation: HW, JW, and XZ. Writing—review and editing: YX and PL. Supervision: QC, YX, and PL. All authors contributed to the article and approved the submitted version.

## Conflict of Interest

The authors declare that the research was conducted in the absence of any commercial or financial relationships that could be construed as a potential conflict of interest.
